# Internet-based cognitive behavioral therapy for bulimic eating disorders in a clinical setting: Results from a randomized trial with one-year follow-up

**DOI:** 10.1016/j.invent.2022.100598

**Published:** 2022-12-21

**Authors:** Louise Högdahl, Andreas Birgegård, Claes Norring, Joakim de Man Lapidoth, Mikael Andersson Franko, Caroline Björck

**Affiliations:** a Department of Clinical Neuroscience, Karolinska Institutet, Stockholm, Sweden; b Centre for Psychiatry Research, Stockholm Health Care Services, Stockholm, Sweden; c Department of Medical Epidemiology and Biostatistics, Karolinska Institutet, Stockholm, Sweden; d TioHundra AB, Department of Psychiatry, Norrtälje Hospital, Norrtälje, Sweden; e Department of Women's and Children's Health, Akademiska sjukhuset, Uppsala University, Uppsala, Sweden; f Centre for Research and Development, Region Gävleborg, Gävle, Sweden

**Keywords:** Eating disorder, Cognitive behavioral therapy, Internet-based treatment, Randomized trial, Clinical setting, Bulimia nervosa, Bulimic eating disorders

## Abstract

**Background:**

Those who suffer from eating disorders often experience serious impairment in quality of life and the majority never receive treatment. Treatment availability may be increased by implementing methods that demand less resources and are more easy accessible such as internet-based treatments, but knowledge about their effects is still insufficient. The study evaluated effects of two types of internet-based cognitive behavioral therapy and a structured day patient program, the latter being a standard treatment at an eating disorder clinic at the time for the study.

**Methods:**

150 participants with bulimic eating disorders randomized to two types of internet based treatments (one pure online treatment and one based on a self-help guide in book-format) or an intensive 16-week day patient program. The number of participants that started treatment was 120 of which 98 in internet treatment and 22 in the day program. Outcome assessments were carried out at baseline, post treatment, and at one-year follow-up.

**Results:**

All treatments were associated with significantly improved eating disorder pathology, self-image, and clinical impairment. Although the day program generally showed larger effects, only one significant difference found was in diagnostic remission post treatment; 51 % of the participant was in remission in internet treatment and 88 % in the day program. At one-year follow-up, participants in the internet treatments had continued to improve, whereas in the day patient program the effect sustained. Internet treatment had a 36 % drop out rate, there were no dropouts found in the day program.

**Conclusions:**

All treatments were comparable in effect at follow-up, suggesting that internet treatment is a conceivable alternative to standard treatment. Internet treatment in a book-based format was also equally effective as a pure online format. Internet delivered cognitive behavioral treatment forms can make important contributions to achieve increased access to treatment for patients with bulimic eating disorders. Future research and clinical implications for internet delivered treatments in eating disorder services are discussed.

**Clinical trial registration:**

ISRCTN registry https://www.isrctn.com/ISRCTN44999017. The study was registered retrospectively.

## Highlights

• Two internet-based cognitive behavioral therapy (ICBT) treatments were compared to an intensive day-patient treatment in patients with bulimic eating disorders in a clinical setting.
• The ICBT treatments were similar in effect, and although the day program generally showed larger effects, only diagnostic remission was significantly different post treatment; 51% in ICBT and 88% in the day program.
• At one-year follow-up, participants in the internet treatments had continued to improve, whereas in the day patient program the effect was sustained.
• All treatments were comparable in effect at follow-up, suggesting that internet treatment may be a more accessible alternative to standard treatments.

## 1 Background

The majority of those with eating disorder (ED) never receive treatment and fewer get access to psychotherapy, likely due to barriers such as shame, fear of stigma, and limited availability (Aardoom et al., 2017; Aardoom et al., 2013; Ali et al., 2017). Also, both sufferers and their relatives tend to experience marked impairment in quality of life, and economic costs of EDs are substantial (Stuhldreher et al., 2012). Cognitive behavior therapy (CBT) is the preferred treatment for adult bulimia nervosa (BN) (Waller, 2016; Mulkens and Waller, 2021; Fairburn et al., 1993), typically lasting about 20 weeks and comprising three stages: behavioral change, cognitive restructuring, and relapse prevention. CBT significantly reduces ED symptoms (Svaldi et al., 2019), and while most studies have been efficacy trials, results may generalize from research to the clinic (Waller et al., 2014; de Jong et al., 2018). However, the limited availability and barriers to seek treatment call for additional options (Agras et al., 2017).

Internet-based CBT (ICBT) may decrease barriers to care, be more accessible, and more available compared to CBT (Aardoom et al., 2016; Machado and Rodrigues, 2019). NICE guidelines state that CBT-based guided self-help should be the firsthand treatment option for BN and binge eating disorder (BED; National Institute for Health and Care Excellence, 2017), and internet-based treatments seem here to stay (Aardoom et al., 2016; Loucas et al., 2014). A meta-analysis found that such interventions can successfully reduce ED symptoms (Taylor et al., 2020), and a review found effects comparable to face-to-face treatments and stable outcomes (Dölemeyer et al., 2013). Another found large effects, especially for binge eating and global ED symptoms such as maladaptive cognitions, at both post and follow-up, whereas results for purging were more mixed (Aardoom et al., 2013). A more recent review showed that 22.1 to 46.5 % follow-up abstinence from binge eating and compensatory behaviors, significantly superior to waiting-list (Pittock et al., 2018). A review of 50 trials of manualized self-help found that guidance improved adherence and outcome in BN, and that guidance by mental health specialists is more effective than that by non-specialists (Beintner et al., 2014). Patients have been found to be generally accepting of the treatment format (Agras et al., 2017). An intriguing pattern is also that improvement may continue in ICBT after termination of treatment (Zerwas et al., 2016; Wagner et al., 2013; Linardon and Wade, 2018). The cited reviews and meta-analyses have included studies on the specific forms of ICBT used in the present study; SALUT-BN (see below) has shown positive results (Carrard et al., 2011a), and likewise the bibliotherapy-based treatment (Ghaderi and Scott, 2003).

However, while a number of studies support ICBT, others present a less positive picture. One review found that while internet-based treatments for EDs showed positive effects, confidence in the effect estimates was low and the value of these treatments was judged as uncertain (Loucas et al., 2014). Further, two recent studies found no clear evidence of ICBT being more cost-effective than CBT (König et al., 2018; Watson et al., 2018), and dropout from ICBT may be higher than in CBT (Pittock et al., 2018; Linardon et al., 2018). Some authors also cite a lack of evidence to show that ICBT has positive effects on bulimic ED-behavior (Pittock et al., 2018). However, most seem to agree that conclusions are not definitive (Aardoom et al., 2016; Loucas et al., 2014; Dölemeyer et al., 2013; Pittock et al., 2018; Linardon and Wade, 2018; Watson et al., 2018; Schleg et al., 2015; Watson et al., 2017), and long-term follow-up has rarely been reported (Schleg et al., 2015).

The current study is randomized trial (Controlled-trials.com/ISRCTN44999017) at a specialist clinic in Sweden, comparing two types of ICBT and an intensive day patient program (DPP) for BN and similar EDs in terms of treatment outcome post and at one-year follow-up. While significantly more intensive, DPP interventions of various formats have been established as a common or standard treatment regimen in ED, including BN, and were therefore judged to be an appropriate comparison (Eshkevari et al., 2022). DPP involved several hours of treatment contact, including meal training, every weekday for 16 weeks, and was therefore many times more intensive, and involved a range of treatment types and modalities, compared to ICBT (see Method). Therefore, the hypothesis was that DPP would be more effective than ICBT since at face value, more treatment was provided. It is important however to investigate whether less intensive and less costly interventions may bestow meaningful benefit. We also explored and expected that ICBT would show similar results to face-to-face CBT as reported in the literature, and that there would be no significant differences between the two types of ICBT in treatment outcome. The former comparison is of interest since guided self-help is a recommended first choice treatment for BN, and generally speaking considerably cheaper. The latter comparison is important since it speaks to whether the content and remote format of the treatments (guided bibliotherapy vs. “Salut BN” interactive online treatment; see below) are sufficient to carry effects or whether the interactive online format, and concomitant license cost (see below), of Salut BN were associated with additional benefit.

## 2 Method

### 2.1 Participants

Recruitment of 150 participants occurred from October 2009 through February 2013 from the specialized clinic Stockholm Center for Eating Disorders (henceforth called the clinic), and patients were admitted through self-enrolment or referral. Inclusion criteria were DSM-IV diagnosis BN or BN-like ED not otherwise specified (EDNOS example 3; “subthreshold BN”; American Psychiatric Association, 2000). These diagnoses correspond to the DSM-5 BN and BN-like other specified feeding and eating disorders (OSFED) (American Psychiatric Association, 2013). BED was included if there was a history of recurrent inappropriate compensatory behavior during the past year. Since treatment included focus on cessation of compensatory behaviors (including regular fasting) BED was otherwise excluded. Further inclusion criteria were Swedish speaking, ≥18 years of age, and body mass index (BMI) 17.5–34. Exclusion criteria were ongoing ED treatment, severe symptoms of depression, anxiety, or obsession/compulsion (maximum score of 15, 15, and 14 respectively on the Comprehensive Psychopathological Rating Scale self-assessment for Affective syndromes (CPRS-S-A, described below)), abuse of alcohol or psychoactive drugs, psychosis, suicide plans or suicide attempt within the past year, and previous participation in ICBT or DPP. Ethics approval was granted by the Stockholm Ethical Review Board (2008/669-31/4).

### 2.2 Design and procedure

The study was a randomized trial with assessments pre, post, and follow-up one year after end of treatment. Those who fulfilled criteria were given written and oral information, asked to participate, and given one week to consider. If they declined, they were offered standard assessment and individualized treatment at the clinic. Randomization was done by random number generator using a pocket calculator (0–0.3299/0.33–0.6699/0.67–1). Participants were then assigned to a therapist appointment where they were informed about treatment allocation. Those who were allocated to ICBT started treatment in connection with the appointment, whereas those allocated to the day program started on a later, fixed date.

Primary outcomes were ED diagnosis and self-assessed ED symptoms. Secondary outcomes were self-image and clinical impairment. Guidelines for executing and reporting internet intervention research (Proudfoot et al., 2011) have been followed as far as possible.

**Fig. 1:**
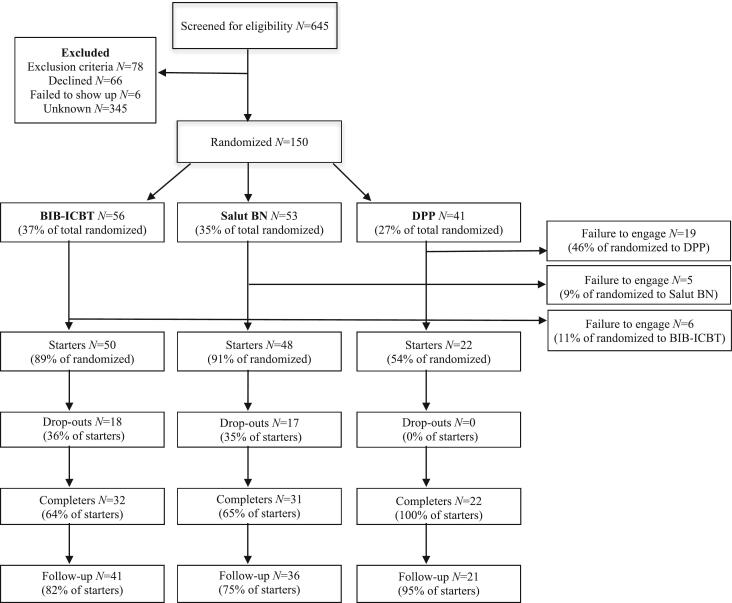
Study inclusion flow chart.

Recruitment of 150 participants took about 40 months, the first 120 during 33 months. Due to time- and organizational reasons an attempt was made to speed this up by removing DPP as a possible allocation. This decision was based on the experience that many were hesitant to enter the trial because there was a mandatory sick-leave during DPP, and also that many who were allocated to DPP ultimately failed to start treatment. Due to the alteration, the last 30 participants were only randomized to one of the two ICBT. However, this change sped up the process only marginally, from 3.6/month to about 4.3/month. Due to administrative changes at the clinic, the study also suffered difficulties in following the participant flow. During the first 29 months, 468 patients were screened, 212 (41 %) of them were not randomized for unknown reasons. Of the remaining 256 patients, 66 (26 %) declined, 78 (30 %) did not fulfil criteria, 6 (2 %) failed to show up, and 106 (41 %) were randomized. Thus, an estimated total of 645 patients were screened for eligibility during the full inclusion period. The participant flow, based on this estimation, is shown in Fig. 1.

### 2.3 Treatment conditions

#### 2.3.1 Internet based cognitive behavior therapy (ICBT)

The two types of ICBT; BIB-ICBT and Salut BN, were similar in several respects. Maximum length was 24 weeks, weekly internet-based asynchronous therapist contact, two face-to-face meetings, and the focus areas 1) behavioral modification and psychoeducation, 2) cognitive restructuring, and 3) relapse prevention. Treatment required approximately 10 min/week/patient for the therapist, as well as 2 h/patient for the face-to-face appointments. Thus, therapist time/patient was about 6 h. The therapist's main role was to support and motivate the participant to actively engage in treatment. There were four therapists, whereof three female and one male. Three were licensed psychologists, and one was a behavioral scientist with psychotherapist training. All had training in CBT and all conducted both ICBT treatments. ICBT was only accessible for patients if they participated in the current study, thus it was not one of the clinic's standard treatments.

Each ICBT also had unique features: BIB-ICBT was bibliotherapy based on a Swedish translation of the manual “Overcoming Binge Eating” (Fairburn, 2003) with six treatment modules: 1) Getting started, 2) Regular eating, 3) Alternatives to binge eating, 4) Problem solving, 5) Dieting and food avoidance, and 6) Relapse prevention. Therapists used a manual (Fairburn, 1999) and communication occurred through a secure platform. BIB-ICBT was evaluated in a previous pilot study with significant small-to-large effect reduction of ED symptoms (Högdahl et al., 2013).

Salut BN (http://www2.salut-ed.org/demo/; 25/11/19) is a CBT-based online program developed by Net Union within the European research project “Salut” (https://www.netunion.com/; 25/11/19). It has seven modules: 1) Motivation, 2) Self-observation, 3) Behaviour modification, 4) Problem solving, 5) Cognitive restructuring, 6) Assertiveness, and 7) Relapse prevention. Patient-therapist communication occurred within the system. Salut BN has been evaluated in several studies (Wagner et al., 2013; Carrard et al., 2011b; Carrard et al., 2006; Fernández-Aranda et al., 2009; Liwowsky et al., 2006; Nevonen and Broberg, 2006a) with significant improvement in ED symptoms and general psychopathology, although results varied between clinics. Salut BN had a license fee and was therefore more expensive for the clinic than BIB-ICBT.

Dropouts were defined as participants who started treatment but ended prematurely, against therapist recommendation or without explanation. Although most were considered completers, only 20 % completed all treatment steps. This compares to two studies that reported rates of 18 % (Nevonen and Broberg, 2006b) and 37 % (Watson et al., 2017).

#### 2.3.2 Day patient program (DPP)

DPP, a standard treatment at the clinic, was a 16-week intensive group treatment influenced by psychodynamic theory and featuring group therapy and individual therapy, daily meals, body knowledge, psychoeducation, psychodrama, discussions on gender roles, and art therapy. In focus were symptoms, self-image, and relationships. Each group had eight members that participated 3.5 h each weekday. There was a reunion 6–8 weeks after end of treatment. Relatives were regularly invited to be informed about the treatment process. Prior to treatment start participants had three individual motivational sessions and signed a contract stating that the goals of treatment are to break destructive eating patterns, normalize eating and weight, strive to comprehend that the symptoms are a way to communicate, explore what the underlying messages are, enable a realistic self-image, work with personal growth, and focus on interactions with others. It also stated that the participant should be motivated and engage actively in treatment, that daily attendance was of great importance, and that it was mandatory to eat the food served. The staff was a multidisciplinary team. DPP was evaluated in the same pilot study as BIB-ICBT (Högdahl et al., 2013), and showed significant moderate to large reduction of ED-symptoms.

### 2.4 Assessments

As a standard routine at the clinic all patients were initially assessed with Stepwise (Birgegård et al., 2010), a computerized system including several clinical- and self-assessments described below.

#### 2.4.1 Clinical assessments

The Structured Eating Disorder Interview (SEDI) was used to diagnose EDs according to DSM-IV, and diagnostic remission at post treatment and follow-up, defined as not fulfilling criteria for an ED. The SEDI is a structured diagnostic interview containing up to 30 questions. Good concordance with the gold standard Eating Disorder Examination interview (Cooper et al., 1989) has been shown in a previous validation (Kendall's tau-*b* = 0.69, *p* ≤ .0001; de Man Lapidoth and Birgegård, 2010).

The Structured Clinical Interview for DSM-IV Axis I Disorders (SCID-I; First et al., 2002) was used to diagnose comorbid psychiatric disorder; interrater reliability has been shown to be moderate to excellent (Lobbestael et al., 2011).

Information about previous ED treatment, duration of illness, occupation etc. was registered in the national quality register for ED treatment, Riksät (https://riksat.registercentrum.se). At follow-up, questions were asked about participation in further ED treatment, psychiatric or medical treatment for any other condition, or experience of major life-events.

#### 2.4.2 Self-assessments

The Eating Disorder Examination Questionnaire; EDE-Q (Fairburn and Beglin, 1994), version 4.0 was used for assessment of ED symptoms, and the global scale was computed for use here, as well as presence and frequency of disturbed eating- and compensatory behaviors. The EDE-Q has 36 items that are rated on a 7-step scale and asks about the past 28 days. Internal consistency and discriminative validity have been shown to be good (Berg et al., 2012), with satisfactory concurrent validity (Fairburn and Beglin, 1994), temporal stability (Mond et al., 2004), and test-retest reliability (Luce and Crowther, 1999). The Swedish version has shown acceptable internal consistency (Mantilla and Birgegård, 2015).

The Structural Analysis of Social Behavior (SASB; Benjamin, 1974) Intrex introject was used for assessment of self-directed behaviors in 36 statements that are scored on a 10 step Likert scale. Based on these ratings, an “Affiliation” vector was computed that corresponds closely to self-esteem. SASB has good content-, construct-, concurrent-, and predictive validity (Benjamin et al., 2006).

Clinical Impairment Assessment (CIA) measures impairment secondary to ED in 16 items re-ported on a 4-point Likert scale. It focuses on the past 28 days, and has been shown to have high levels of internal consistency, test-retest reliability, discriminant- and construct validity, and sensitivity to change (Bohn et al., 2008).

The Comprehensive Psychopathological Rating Scale self-assessment for Affective syndromes (CPRS-S-A) measures psychiatric symptoms during the past three days on a 19 item seven-step scale in three domains; depression, anxiety, and obsession-compulsion. It is considered a useful and reliable measure for rating of symptoms in outpatients (Mattila-Evenden et al., 1996).

### 2.5 Statistical analysis

Data were analyzed with SPSS for Mac (v.22) and R. For comparisons of categorical data Chi square was used with phi effect sizes, small ≥0.10, moderate ≥0.30, and large ≥0.50. *t*-Tests were used for comparisons of continuous data with Cohens *d* effects small ≥0.20, moderate ≥0.50, and large ≥0.80. Power analysis showed beta = 0.90 for 40 participants in each group. Correlations were Pearson's *r* judged as weak ≥0.10 moderate ≥0.30, or strong ≥0.50. Between-group comparisons of interaction effects between time and treatment type on EDE-Q total, self-image, and impairment were analyzed using mixed linear models with an interaction between time point and treatment group adjusted for age. The repeated measure structure was handled by including an unstructured covariance matrix with heterogeneous variances for each participant. All models were fitted with function lme from package nlme and results were calculated as estimated means using package emmeans in R.

Since binge eating, purging and exercise were count data, these variables were analyzed using mixed negative binomial models with a zero inflation term to account for the high frequency of zero counts. Both the conditional model and the zero inflation model included an interaction between time point and group adjusted for age. All models were fitted with function glmmTMB from package glmmTMB, and results were calculated as estimated means using package emmeans in R. Alpha = 0.05 was used for all analyses. There were no significant differences between those who were randomized to all three treatments and those who only were randomized to the ICBT arms in any of the measures (all *p* > .05).

## 3 Results

### 3.1 Comparisons of ICBT groups

There were no significant differences between BIB-ICBT and Salut BN in BMI, age, ED-diagnosis, EDE-Q total, binge eating, purging, exercise, clinical impairment (CIA), depression, anxiety, obsession/compulsion (CPRS-S-A scales), self-image (SASB), drop-out rate, or time spent in treatment either post or at follow-up, including occurrence of further treatment during the follow-up year. There-fore, BIB-ICBT and Salut BN are henceforth analyzed and presented combined as “ICBT”.

### 3.2 Adherence and treatment delay

Of the 150 randomized participants, 120 (80 %) started treatment. Failure to engage (FTE) was defined as complete initial assessment and consent to participate but failure to start treatment. A substantial 46 % allocated to DPP were FTE, whereas in ICBT 10 % were FTE (different at χ^2^ = 24.47, *p* < .001, phi = 0.404, a moderately strong effect). There were no significant differences between starters and FTE in any of the measures (all *p* > .097), and FTE was excluded from further analysis.

Of the 120 starters, 85 (71 %) participants were considered completers; 64 % in ICBT and 100 % in DPP (χ^2^ = 11.09, *p* < .001, phi = 0.304, a moderate effect). ICBT completers were defined as being actively engaged in treatment until reaching sufficient symptom reduction based on a clinical evaluation and decided in collaboration with the patient, reaching the maximum time of 24 weeks, or finishing all treatment steps. ICBT average length was 17 weeks (*SD* = 7.6), and drop-out rate was 36 %, There were no drop-outs in DPP. ICBT Completers participated on average 21.3 weeks (*SD* = 4.31) and drop-outs 10.2 weeks (*SD* = 7.15), a significant difference (*F* = 92.42, *p* < .001). Frequency of further treatment during the follow-up year was not significantly different (*χ*2 = 0.400, phi = 0.064, *p* = .674) between completers (52 %) and drop-outs (46 %). Pre-treatment data for all completers and drop-outs has been reported previously (Högdahl et al., 2016).

ICBT participants started treatment on average 4.8 (*SD* = 3.3) weeks after initial assessment. In DPP, treatment started on average after 11.8 (*SD* = 7.6) weeks, which was significantly longer (*F* = 44.70, *p* < .001). Time from initial assessment to treatment start did not correlate with outcome for either ICBT or DPP (all *p*s > .375, all *r*s < 0.173). Further ED treatment during the follow-up year was common with 50 % in both ICBT and DPP. Eight participants in ICBT participated in DPP during the follow up year. Results were not markedly different when these participants were omitted from follow-up analyses.

### 3.3 Participant characteristics

Baseline characteristics and comparisons of ICBT and DPP are shown in Table 1. All but one were female. Any comorbidity was found in 64 % whereof 62 % in ICBT and 73 % in DPP, not a significant difference (*p* = .463, phi = 0.085). EDE-Q and CIA scores were high, and SASB self-image scores low, indicating that participants had a high ED-symptom load, impaired functioning, and a negative self-image. Although binge eating is a core symptom, 21 % in ICBT and 27 % in DPP scored zero at baseline. A possible explanation is that binge eating is difficult to assess; one study found that patients with BN and BED reported a lower number of episodes on a self-assessment than in a structured interview (Birgegård et al., 2014). Also, symptoms vary over time and fluctuations are typical (Williams et al., 2012) and the EDE-Q covers a time frame of one month whereas diagnostic procedures considered three months as specified in the DSM. No significant baseline differences were found between ICBT and DPP except for higher purging frequency in DPP.

**Table 1:** Baseline characteristics of participants in ICBT and DPP.

Participant characteristics	ICBT	DPP	*p*
ED diagnosis			.631
Bulimia	60 (61 %)	15 (68 %)	
EDNOS	38 (39 %)	7 (32 %)	
Comorbidity			
Depression	29 (30 %)	10 (45 %)	.355
Anxiety	49 (50 %)	12 (55 %)	.815
Dependency	5 (5 %)	2 (9 %)	.611
Age, years	27.3 (7.28)	26.5 (6.35)	.645
Duration, years	11.7 (8.55)	9.9 (7.17)	.346
BMI	23.2 (3.20)	24.4 (4.52)	.146
SASB self-image	−2.8 (34.60)	−9.0 (26.73)	.432
EDE-Q total	3.7 (1.12)	4.1 (0.83)	.093
Binge eating frequency	8.5 (8.68)	9.2 (8.48)	.720
Purging frequency	7.5 (10.08)	14.1 (18.24)	.022
Exercise frequency	8.6 (9.28)	8.4 (11.45)	.936
CIA	25.7 (9.92)	28.2 (7.30)	.262

Note. ICBT *N* = 98, DPP *N* = 22. ED diagnosis refers to DSM-IV diagnosis obtained based on The Structured Eating Disorder Interview. EDNOS includes 93 % BN-type and 7 % BED. Comorbidity refers to presence of DSM-IV diagnosis obtained using The Structured Clinical Interview for DSM-IV Axis I Disorders. Depression includes major depression and not otherwise specified (NOS).

Anxiety includes agoraphobia, panic disorder, obsessive-compulsive disorder, posttraumatic stress disorder, social phobia, specific phobia, generalized anxiety disorder, and anxiety NOS. Substance abuse includes both alcohol- and drug abuse. *ED* eating disorder; *BMI* body mass index; *SASB* Structural Analysis of Social Behavior; *EDE-Q* Eating Disorder Examination Questionnaire; *CIA* Clinical Impairment Assessment.

### 3.4 Primary and secondary outcomes

DPP was more effective than ICBT regarding diagnostic remission at post treatment. However, at one year follow-up remission had increased in ICBT and decreased in DPP, and treatments were no longer significantly different (Table 2).

**Table 2:** Remission rates of ED diagnosis at post and at one year follow-up.

Outcome	ICBT	DPP	*χ*^2^	*phi*	*p*
Post treatment	51 %	88 %	8.153	0.29	.006
Follow-up	75 %	70 %	0.197	0.05	.772

Note. ICBT post *N* = 83, DPP post *N* = 17. ICBT follow-up *N* = 64, DPP follow-up *N* = 20.

In both treatments there were significant improvement from pre to post in EDE-Q, self-image, and impairment. In ED behaviors, binge eating improved significantly in both treatments, purging did so in DPP but not in ICBT, and no significant effect was found for exercise. Effect sizes were larger in DPP. From post to follow up however there were larger improvements in ICBT overall, and from pre to follow-up there were significant improvements in all outcomes for ICBT, but although effect sizes were comparable between treatments, statistical significance was not reached for binge eating, purging, or exercise in DPP (Table 3, Table 4).

**Table 3:** Self-assessed cognitive ED symptoms, self-image, and clinical impairment at pre, post, and follow-up, and t, p and effect size (ES; calculated as difference between means) for pre-post and pre-follow-up contrasts. Note that only effect size is shown for post to follow-up.

Variable	Measure	Pre	Post	FU	Pre-post			Post-FU	Pre-FU		
	Group	*M* (*SE*)	*M* (*SE*)	*M* (*SE*)	*ES* (*SE*) pre-post	*t* pre-post	*p* pre-post	*ES* (*SE*) post-FU	*ES* (*SE*) pre-FU	*t* pre-FU	*p* pre-FU
EDE-Q total	ICBT	3.7 (0.11)	2.4 (0.14)	2.0 (0.16)	1.3 (0.15)	8.40	<.001	0.40 (0.17)	1.68 (0.18)	9.30	<.001
	DPP	3.9 (0.18)	1.8 (0.29)	1.8 (0.31)	2.1 (0.31)	6.57	<.001	0.01 (0.34)	2.07 (0.34)	6.09	<.001
Self-image	ICBT	−2.7 (3.2)	24.4 (4.1)	39.2 (4.2)	−27.1 (3.7)	−7.37	<.001	−14.8 (4.4)	−41.9 (4.6)	−9.10	<.001
	DPP	−4.8 (5.1)	28.3 (8.1)	27.7 (8.0)	−33.1 (7.6)	−4.34	<.001	0.6 (8.5)	−32.6 (8.6)	−3.78	<.001
Impairment	ICBT	26.1 (0.9)	17.0 (1.3)	12.3 (1.2)	9.2 (1.4)	6.57	<.001	4.6 (1.6)	13.8 (1.3)	10.46	<.001
	DPP	26.5 (1.5)	12.6 (2.9)	9.9 (2.3)	13.9 (3.0)	4.72	<.001	2.7 (3.2)	16.7 (2.5)	6.67	<.001

Note. EDE-Q = Eating disorders examination questionnaire; ICBT = internet-based cognitive behavior therapy; DPP=Day patient program; FU = follow-up; *ES* = effect size. Data is presented for participants with both pre, post, and follow-up measures; ICBT *N* = 63, DPP *N* = 16.

**Table 4:** Mean frequencies and standard errors for self-assessed behavioral ED symptoms at pre, post, and follow-up, and t, p and effect size (calculated as a quotient of post/pre) for the pre-post and pre-follow-up contrasts. Note that only effect size is shown for post to follow-up.

Variable	Measure	Pre	Post	FU	Pre-post	Pre-post		Post-FU	Pre-FU		
	Group	*M* (*SE*)	*M* (*SE*)	*M* (*SE*)	*ES* (*SE*)	*t* pre-post	*p* pre-post	*ES* (*SE*)	*ES* (*SE*)	*t* pre-FU	*p* pre-FU
Binge eating	ICBT	10.2 (1.0)	6.8 (1.1)	4.3 (0.9)	1.51 (0.27)	2.33	.020	1.57 (0.37)	2.37 (0.50)	4.08	<.001
	DPP	12.6 (2.0)	2.9 (1.6)	6.1 (2.6)	4.34 (2.43)	2.62	.009	0.47 (0.32)	2.06 (0.91)	1.63	.104
Purging	ICBT	11.8 (1.8)	8.2 (1.9)	4.2 (1.0)	1.44 (0.29)	1.82	.070	1.96 (0.50)	2.83 (0.66)	4.49	<.001
	DPP	15.6 (3.3)	1.9 (1.3)	5.7 (3.0)	8.21 (5.36)	3.22	.001	0.33 (0.27)	2.73 (1.48)	1.84	.066
Exercise	ICBT	12.1 (1.1)	9.3 (1.3)	8.1 (1.4)	1.31 (0.19)	1.84	.067	1.14 (0.23)	1.49 (0.27)	2.19	.030
	DPP	12.1 (1.8)	6.2 (3.2)	7.9 (3.0)	1.94 (0.99)	1.29	.198	0.79 (0.48)	1.52 (0.60)	1.07	.286

Note. ICBT=internet-based cognitive behavior therapy; DPP=Day patient program; FU = follow-up; ES = Effect size. Data is presented for participants with both pre, post, and follow-up measures; ICBT *N* = 63, DPP *N* = 16.

There were no significant Time (3 measurements) X Treatment (2 groups) interaction effects in EDE-Q total (χ^2^[2] = 4.85, *p* = .088), self-image (χ^2^[2] = 2.57, *p* = .277), impairment (χ^2^[2] = 2.42, *p* = .298), binge eating frequency (χ^2^[4] = 4.13, *p* = .389), or exercise frequency (χ^2^[2] = 3.36, *p* = .500), but there was a significant interaction in purging frequency (χ^2^[4] = 10.57, *p* = .032) suggesting that DPP decreased significantly more. Pairwise contrasts between ICBT and DPP at each time point showed one significant difference: DPP had significantly lower purging frequency at post treatment (likelihood ratio = 4.29 [2.88], *p* = .031). No other significant differences were found (all *p*s > .07).

Baseline abstinence in ICBT was 21 % for binge eating, 43 % for purging, and 32 % for exercise. In DPP it was 27 % for binge eating, 36 % for purging, and 41 % for exercise. Reductions and abstinence rates are shown in Table 5.

**Table 5:** Percent reduction of frequencies, and proportion abstinent from, binge eating and compensatory behavior during the past 28 days.

Variable	Measure		
	Group	Pre-post frequency reduction	Pre-follow-up frequency reduction
Reduction			
Binge eating	ICBT	58 %	75 %
	DPP	89 %	78 %
Vomiting	ICBT	28 %	74 %
	DPP	93 %	86 %
Exercise	ICBT	51 %	66 %
	DPP	86 %	75 %


Variable	Measure		
	Group	Proportion abstinent	Proportion abstinent
		Post	Follow-up
Abstinence			
Binge eating	ICBT	56 %	63 %
	DPP	76 %	74 %
Vomiting	ICBT	63 %	67 %
	DPP	82 %	79 %
Exercise	ICBT	62 %	73 %
	DPP	88 %	79 %
Total	ICBT	38 %	44 %
	DPP	65 %	63 %

Note. Abstinence refers to proportion of participants that have scored a zero with respect to the last 28 days. Total refers to abstinence from both binge eating and vomiting.

## 4 Discussion

Based on dose-response reasoning, we expected that the more treatment-intense DPP would outperform ICBT. This was supported regarding diagnostic remission post treatment, but not statistically in other respects except for the significant Time X Treatment interaction in purging frequency. While post-treatment effects in self-assessments and diagnostic remission were larger in DPP, there was continued improvement to follow-up in ICBT, but stability or reduction in DPP, and at follow-up the groups were more similar than at post. Whether intensity per se or theoretical orientation of the treatments, or both, may contribute to these patterns could usefully be studied in future research.

Cognitive ED symptoms (EDE-Q) and clinical impairment (CIA) were significantly reduced with large effects for both treatments, and SASB self-image also improved significantly with similar moderate effects. The lack of difference in the latter is noteworthy since self-image was a main treatment focus in DPP but not in ICBT.

The pattern that ICBT outcome improves over time has, as mentioned, been observed before (Zerwas et al., 2016; Wagner et al., 2013; Linardon and Wade, 2018). In the present study, further treatment during follow-up was not significantly different and therefore unlikely to account for the differential continued improvement. This may suggest that guided self-help takes longer to achieve its full effect, but the autonomy and high own responsibility for improvement may be empowering. In contrast, the high level of structure and “pause” in normal life activities in DPP may to a lesser extent enable resumption of daily life while maintaining treatment gains.

Significantly more participants randomized to DPP were FTE, possible because some consented hoping to be randomized to ICBT and dropped out when that did not occur. At the time, inclusion in the study was the only way to obtain ICBT, whereas DPP was a standard treatment. DPP required full sick-leave, and a daily attendance of 3.5 h throughout treatment, which may have been discouraging to some. Full-time sick-leave during DPP was the policy of the treatment staff at the clinic, but day treatment is in principle possible to combine with, e.g., part-time employment or studies, and other policies regarding this issue could affect acceptability and outcomes in day treatment formats.

In contrast, there were no drop-outs in DPP, but 36 % in ICBT, in another study shown to be predicted by personality and therapist factors (Högdahl et al., 2016). Another study (Watson et al., 2017) found that being randomized to a non-preferred format predicted drop-out, which may have occurred in ICBT, whereas in DPP those who finally did engage were highly motivated. That is, opting out of treatment may have depended on partly similar factors but occurred at different times, due to the higher intake threshold into DPP, e.g., the format with several motivational sessions which were also an opportunity for clinicians to gauge motivation. Also, DPP included a supporting session for relatives, mandatory sick-leave, signing of a contract, and focus on group processes, features that may have helped prevent drop-out. In ICBT however, the threshold for drop-out was lower because of its independent and low-intensive nature. Thus, when taking both FTE and dropout into account, treatment acceptability of DPP and ICBT may be said to be roughly similar. The drop-out rate in ICBT was comparable to other studies (Dölemeyer et al., 2013; Pittock et al., 2018).

We expected that ICBT would be equally effective as CBT, and overall this was confirmed. Diagnostic remission in the current study was 51 % post and 75 % at follow-up, similar to those often reported for CBT, e.g. about a 40–50 % (Anderson and Maloney, 2001). Pre to post effects were small to large, except for purging that showed close to no effect. A meta-analysis reported large effects for CBT; *d* = 1.19 for binge eating, and *d* = 1.15 for purging (Thompson-Brenner et al., 2003). In the present study, a moderate post-treatment effect for binge eating (*d* = 0.75) became large at follow-up (*d* = 0.93), and there was a moderate follow-up effect for purging (*d* = 0.63). Regarding abstinence, one meta-analysis found about 50 % in BN patients post CBT, and those not abstinent showed an average binge eating frequency of 1.32/week, and purging 1.68/week (Thompson-Brenner et al., 2003). In the current study complete abstinence in ICBT was slightly lower with 38 % post treatment, and 44 % at follow-up. Non-abstinent participants showed an average weekly binge eating frequency of 0.80/week, and 1.53 for purging. At follow-up, binge eating was further reduced to 0.56, and purging also to 0.56. Thus, results in the current study were comparable to CBT regarding diagnostic remission, effects for binge eating, complete abstinence, and frequency of binge eating and purging in non-abstinent participants.

Finally, our assumption that both ICBT treatments would be equally effective was confirmed, in line with another study that found no significant differences between Salut BN and a bibliotherapy type of ICBT, similar to the BIB-ICBT in the current study (Wagner et al., 2013). Taken together, this suggests that delivery mode does not carry the effect, although the license cost associated with Salut BN could be considered a disadvantage.

The initial conclusions from previous ICBT research were predominantly positive, and within a relatively short time internet-based treatments grew popular. Then the pendulum swung and some caution was voiced against the treatment lacking sufficient support, and not being cost-effective. Perhaps the next development is a synthesis of these views; ICBT does generate positive outcome for some patients, and potential benefits outweigh potential risks.

### 4.1 Strengths and limitations

Strengths include the randomized design, the streamlined assessment procedure with validated instruments, and that it was conducted in a clinical environment which increases generalizability. Questions about further treatment during follow-up facilitated evaluation of the actual treatment effect. The partial lack of information on how many patients were not asked to participate although they should have been limits generalizability. Also, our relatively stringent inclusion criteria (e.g., allowing only relatively mild psychiatric comorbidity), established to ensure patient safety in the context of this, at that time, relatively untested treatment, limits generalizability also, and results must be interpreted with this in mind. The change to randomizing to only ICBT for the last 30 participants raises the issue over the definition of a randomized trial, but there were no differences between patients randomized to all three vs. two. Relatively small groups and many cases of missing data also reduces ability to draw firm conclusions. Since DPP was based on a different theoretical orientation, we cannot interpret efficacy based on intensity or theoretical content, and another more intensive CBT-based day patient treatment could have yielded different results. Further, the study was not blinded. Assessments were usually carried out by one of the ICBT-therapists, and only rarely by staff at DPP (however, one of the ICBT-therapists was also staff at DPP) and there is a risk of allegiance effects. Self-assessments are however less dependent on the therapists' evaluation, and possible effects of gratitude, guilt, or consideration of the therapist would presumably have affected ICBT and DPP similarly.

### 4.2 Future studies

Treatment efficacy and effectiveness research need to be further complemented with a cost-effectiveness perspective. In combination with longer follow-up, such research could yield important policy-affecting data concerning what treatments are useful in the context of finite and often diminishing resources. Future research should also examine what type of patient is most likely to benefit from ICBT, and what the most and least potent parts are in order to further improve treatment.

## 5 Conclusions

ICBT, regardless of format, performed similarly over the longer follow-up as an intensive day treatment. ICBT was also comparable to CBT in most outcome measures. Although more research is needed, ICBT should be considered as potential alternative in routine practice.

## Ethics approval and consent to participate

Ethical approval was granted by the Stockholm Ethical Review Board (2008/669-31/4), and all participants provided informed consent.

## CRediT authorship contribution statement

LH was project leader of the study and wrote the manuscript in collaboration with AB, CB, CN, and MAF. AB supervised the later part of the project and wrote the final version of the manuscript together with CB. MAF contributed statistical analyses and consultation, JdML and CN helped design the study and collaborated on data collection, and CB also initiated the study and provided funding together with LH and AB.

## Declaration of competing interest

The authors declare no conflict of interest.

## Data Availability

The data that support the findings of this study are available on request from the corresponding author. The data are not publicly available due to privacy or ethical restrictions.
